# A review of the reproductive system in anuran amphibians

**DOI:** 10.1186/s40851-023-00201-0

**Published:** 2023-02-13

**Authors:** Maribel Méndez-Tepepa, Cuauhtémoc Morales-Cruz, Edelmira García-Nieto, Arely Anaya-Hernández

**Affiliations:** Centro de Investigación en Genética y Ambiente, Universidad Autónoma de Tlaxcala, Autopista San Martín-Tlaxcala Km 10.5, Ixtacuixtla, 90120 Tlaxcala, Mexico

**Keywords:** Anura, Mating systems, Oocytes, Oviduct, Fertilization, Spermatozoa

## Abstract

Reproductive biology is an important topic that is well explored in many vertebrates, but information about frogs’ reproductive mechanisms could be improved. Therefore, this review aims to provide organized and specific information on frog reproduction. First, we developed schemes that illustrate the general information regarding reproductive biological mechanisms in frogs in a specific way. Then, we described the physiological, histological, and morphological mechanisms of each organ of the reproductive system of male and female frogs. Finally, this manuscript may contribute to a broader understanding of anuran reproductive biology. Since, understanding frogs’ reproductive system permits one to make a comparison with reproduction with other anurans.

## Introduction

Different animal groups developed divergent reproductive mechanisms. Amphibians have two sexual reproductive types: internal and external. In this publication, we speak of internal fertilization that involves a diversity of incubation structures that can be dedicated or temporary. There are also significant differences in the levels of development attended by the parents, ranging from larval to juvenile frogs. The main components of the male reproductive system are the testicles and seminiferous tubules, which are associated with fat bodies. Fat bodies play an essential role in producing reproductive hormones [1]. The testicles consist of a seminiferous tubule network [2] that produces spermatozoa [3]. In fact, spermatozoa morphology varies among frog species [4–6]. Meanwhile, the female frog’s reproductive system comprises oviducts and ovaries attached to fat bodies. The ovaries’ fat bodies contribute to the formation of follicles, oocytes, hormones, and yolk [7–9]. The reproductive process begins in the ovaries with the formation of oogonia and oocytes [10]. The oviduct is a tubular organ connected to the ovaries. The oviduct consists of three regions: infundibulum, ampulla, and isthmus. These regions participate in the oocyte’s capture, secretion, and fertilization during its transport to the ovisac. Finally, the ovisac or uterus stores the oocytes for subsequent fertilization by the spermatozoa [11–13].

Following fertilization, embryo development and maintenance vary among species [14]. Some species give birth to developed frogs, while others give birth to larval frogs. In contrast, others have implemented unusual mechanisms for embryo development, such as the utilization of structures such as the stomach, dorsal sac [15], or vocal sac [16] as a gestational organ [17]. Therefore, it is essential to describe all known reproductive mechanisms. Principally, reproduction varies in some frog species, from mating to internal or external fertilization. In particular, this review aims to integrate and describe each participating organ’s physiological, histological, and morphological functions in reproduction. Therefore, we propose reproductive schemes that describe the importance of the different mating behavior until vital reproductive mechanisms lead to the success of their internal or external sexual reproduction.

### Mating systems and reproductive modes in frogs

The amphibians have developed a diverse range of reproductive modes, i.e., strategies related to reproductive behavior and oviposition [18]. Therefore, the oviposition site, oocyte characteristics, clutch-type, size of hatchlings, and parental care are associated with reproductive modes [19]. Anurans have internal and external sexual reproduction, as well as different mate attraction mechanisms. In frogs, the reproductive mechanism initiates when males attract females by producing vocalizations, which are amplified by the vocal sac [20]. In addition to vocalizations, male frogs may use visual signals or secretion of pheromones to attract females [21]. Most males vocalize once they reach sexual maturity; they emit calls to attract mates and maintain territorial boundaries. Meanwhile, adult females emit sounds to alert males about threats or courtship during copulation [22]. In some species, the vocal sac has been coopted temporarily as a gestational organ to incubate eggs and larvae [16]. Histologically, the vocal sac is delimited by epithelium, connective tissue, and a muscle layer [17]. Frogs’ mate attraction behaviors vary widely and can be elaborated differently. For example, *Trachycephalus hadroceps* emits 38,000 calls per night [23]. *Micrixalus saxicola* performs feet-flagging behavior, i.e., displays a bright white vocal sac when vocalizing and emits a visual signal to females when deploying the foot [20]. *Xenopus laevis*, *Pelophylax spp*, and *Trachycephalus spp* emit sounds underwater [24, 25], and *Physalaemus pustulosus* inflates the vocal sac to attract its mate [26]. The canebrake tree frogs (genus *Aplastodiscus*) emit pheromones during courtship. Male *Aplastodiscus perviridis* guide females to the underground nests they have built to lay their eggs [27]. Other genuses (*Hyperoliidae*, *Afrixalu*s, *Heterixalus*, and *Phlyctimantis*) have a gular patch that produces up to 65 volatile compounds, such as sesquiterpenes, alcohols, and macrolides [28]. *Dendropsophus ebraccatus* vocalizes continuously, and then females jump in a zigzag pattern and copulate [25].

**Fig. 1:**
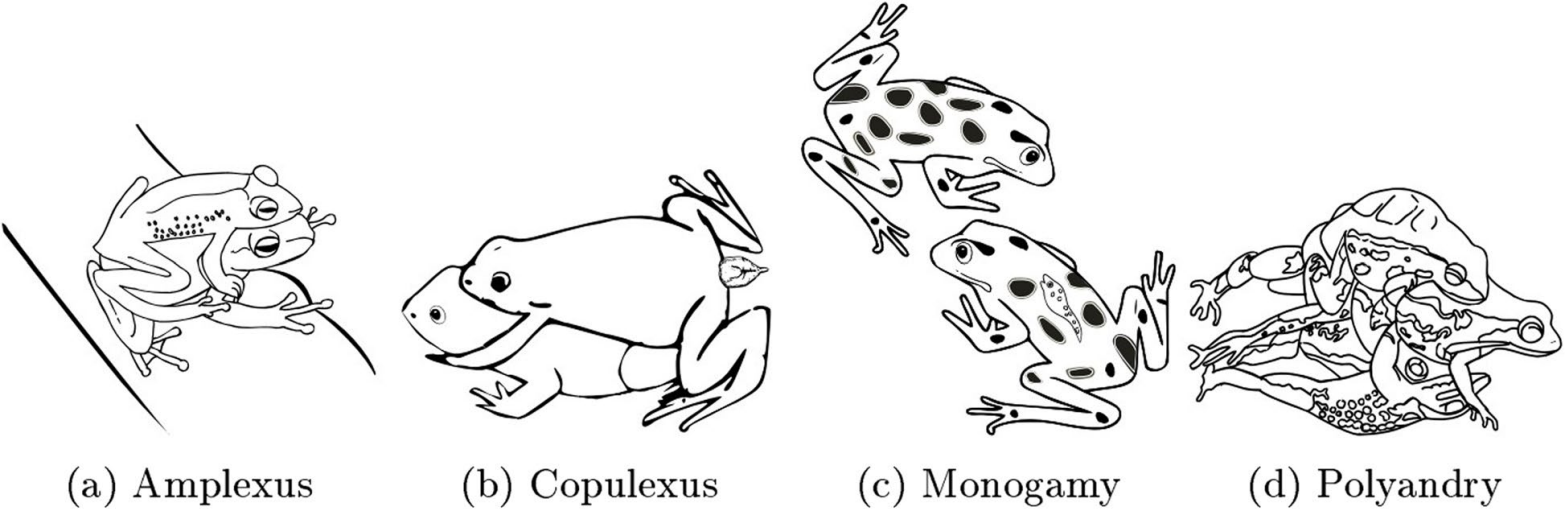
Mating systems and modes in frogs. Amplexus; a male can grasp a female with his front legs by the female’s head, waist, or armpits. This reproductive mode occurs in externally fertilizing species **(a)**. Copulexus; a male clasps the female inguinally rather than axillary to insert its tail. This reproductive mode occurs in internally fertilizing species **(b)**. Monogamy is a mating system where females and males select each other exclusively to couple **(c)**. In contrast, polyandry is when a female mates with two or more different males **(d)**

Amplexus (copulatory embrace) is the reproductive mode exhibited by externally fertilizing species of amphibians [18]. Different types of amplexus have been determined, for example, inguinal, axillary, cephalic, gular, glued, dorsal straddle, head straddle, and loose axillary amplexus [29]. In the amplexus position, the male grasps the female with his front legs by the head, waist, or armpits (Fig. 1a). The duration of amplexus may vary from several hours up to months (e.g., *Atelopus oxyrhynchus*), and can persist throughout the time spent searching for or preparing nests [30]. Then, females may search for suitable oviposition sites such as leaves, puddles, and waterbodies [25]. *Ascaphus truei* is one of the few species of anurans in which fertilization is internal. In these amplexus is replaced by copulexus (a combination of amplexus and copulation), and the grip by the male is inguinal rather than axillary. The tail is used as a copulatory organ and is always inserted during copulation. The sperm transference occurs by extension of the male’s cloaca (Fig. 1b). The tail contains vascularized tissue that becomes engorged and forms a sulcus for passage of sperm when inserted into the female [31]. In *Ascaphus truei* and *Eleutherodactylus jasperi*, sperm can be stored in the female’s oviduct [12, 30].

Some amphibians are socially monogamous; monogamy is a mating system that is characterized by the formation of a pair bond between one male and one female. It often involves increased territorial defense and other types of parental care. Monogamy also implies social interaction between a male and a female frog over a more extended period [32]. *Ranitomeya imitator* is monogamous and performs parental care that is essential for the survival of the tadpoles. In this species, the female selects a single male to oviposit the eggs (Fig. 1c). A week later, the male returns to the oviposition site, carries a tadpole on his back and transports it to a pond where the tadpole will develop [33, 34]. Other species that exhibit this behavior are *R. flavovittata* and strawberry-frog (*Oophaga pumilio*) [35]. On the other hand, polyandry occurs when a female reproduces with more than one male (Fig. 1d). There is a hypothesis that Polyandry ensures the fertilization of the female eggs. In *Chiromantis xerampelina*, females mate with multiple males. Females that mate with up to 12 males are more successful at fertilization [36]. For example, *Proceratophrys goyan* has multiple matings out of the water, and they move to streams to release their eggs [37]. Polyandry is reported in eight species [38], of which only two species (*Crinia georgiana* and *Litoria peronii*) have high reproductive success [39, 40]. The female frogs of *Crinia georgiana*, *Feirana taihangnicus*, and *Rana temporaria* are fertilized by various males [41], one in the dorsal position and the other in the ventral position. Other species, such as *Chiromantis xerampelina* [38], and the Australian quacking frog (*Crinia georgiana*) can mate with as many as five to 12 males [42]. In summary, reproductive strategies in frogs involve the reproductive modes (amplexus and copulexus). Amplexus results in external fertilization, when the male fertilizes the eggs as they are being released by the female. In contrast, internal fertilization is achieved by copulexus, during which the male inserts the tail (“penis”) into the female and deposits the spermatozoa into the oviducts [43, 44]. Generally, the frog species can exhibit a mating system of polyandry or monogamy. Polyandry is associated with multiple ejaculations, sperm transport, and storage, oviposition, and it can also involve parental care [12]. In contrast, monogamy is associated with the exclusive selection of one partner for mating. However, detailed information on reproductive biology still needs to be included, including behavior and mating for many species.

### Female frog reproductive system

#### Ovary and fat bodies

The ovary is composed of two sacs, each of which includes multiple lobes, and each lobe contains thousands of oocytes [45]. In addition, the ovaries have melanophore cells [46] and finger-shaped fatty bodies in the proximal side, attached to the kidneys [47]. Amphibians’ fat bodies are located in the gonads of both males and females. The fat bodies of females are lobular structures that are yellow or orange in color. Histologically, fat bodies are formed by adipose tissue and blood vessels [48]. The fat bodies are involved in the metabolism of gonadal processes [1, 49], mainly in the synthesis and storage of lipids (diglycerides and sterols) [50], fat-soluble vitamins, and esters [51]. Fat bodies also participate in energy supply [52], folliculogenesis, gonadosomatic index (gonad weight/body weight × 100), oxygen uptake [7], yolk production [53], and steroidogenesis. Fat bodies synthesize pregnenolone, progesterone, dehydroepiandrosterone, testosterone, and estrone. Some researchers have determined that the synthesis of progesterone, androstenedione, and testosterone increases during the reproductive season in frogs [54, 55].

**Fig. 2:**
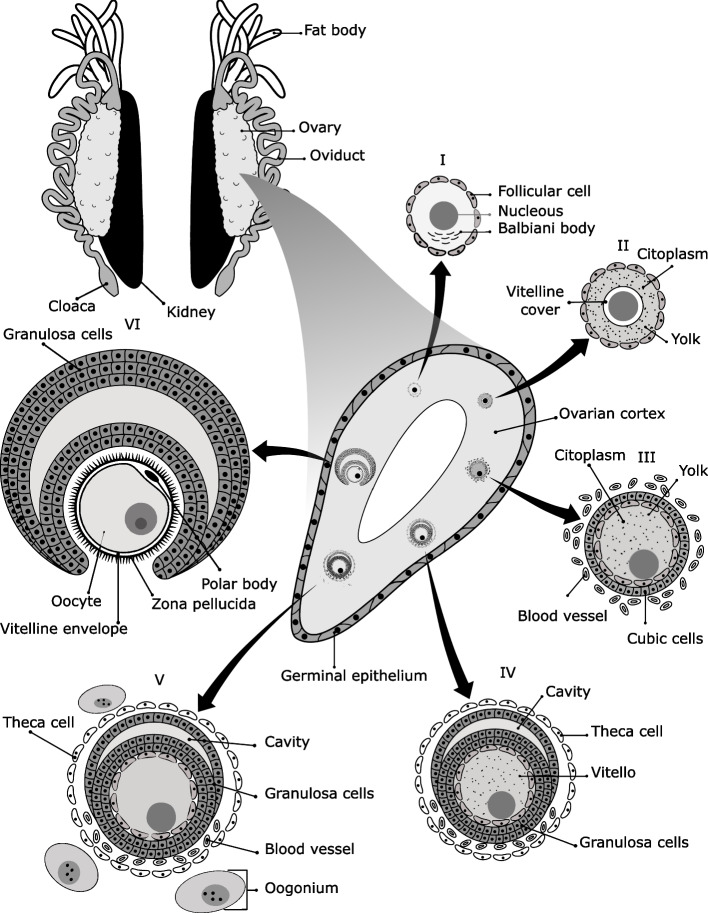
Ovarian morphology and follicular development in frogs. The fat bodies are attached to the ovaries, and the ovaries connect with the oviducts. The ovary is formed by a simple epithelium and ovarian cortex. Follicular development occurs in the ovarian cortex. Stage I. The oocytes are formed by cytoplasm, Balbiani bodies, and the yolk. The follicle is surrounded by the follicular cells. Stage II. Then, the oocytes accumulate yolk and form the acellular envelope. Stage III. Theca and granulosa cells begin to form in the follicle. The height of the cubical follicular cells increases and numerous blood vessels develop. The oocyte accumulates yolk for the later nutrition of eggs. Stage IV. Multiple layers of the granulosa surround oocytes and form a follicular cavity. The blood vessels surround theca cells. Stage V. The zona pellucida and microvilli formation begins on the oocyte’s surface. The oogonia form cellular nests near the follicle. Stage VI. Finally, numerous microvilli, a zona pellucida, and a double vitelline layer surround the oocyte. The follicle finishes its development, and oocytes are ready to be released

Morphologically, the ovaries are composed of the stroma and epithelium. The ovarian stroma is formed by the cortex and medulla, which originates from the peritoneum. The epithelium of the ovary (coelom) derives from the mesoderm and is formed by squamous cells that cover the outside of the ovary [56]. The ovarian epithelium is a layer of flattened cells that form the inner layer of the theca, and granulosa cells (Fig. 2) [57]. Those cells produce reproductive hormones and support ovarian follicles. The granulosa cells are also the main sites of the synthesis of progesterone and estradiol in the ovary. The theca cells are found between the two epithelia in interfollicular regions. Theca cells surround the layer of follicle cells and are formed by collagen, blood vessels, and fibroblasts. The fibroblasts are flat cells that synthesize the extracellular matrix and collagen [56].

**Fig. 3:**
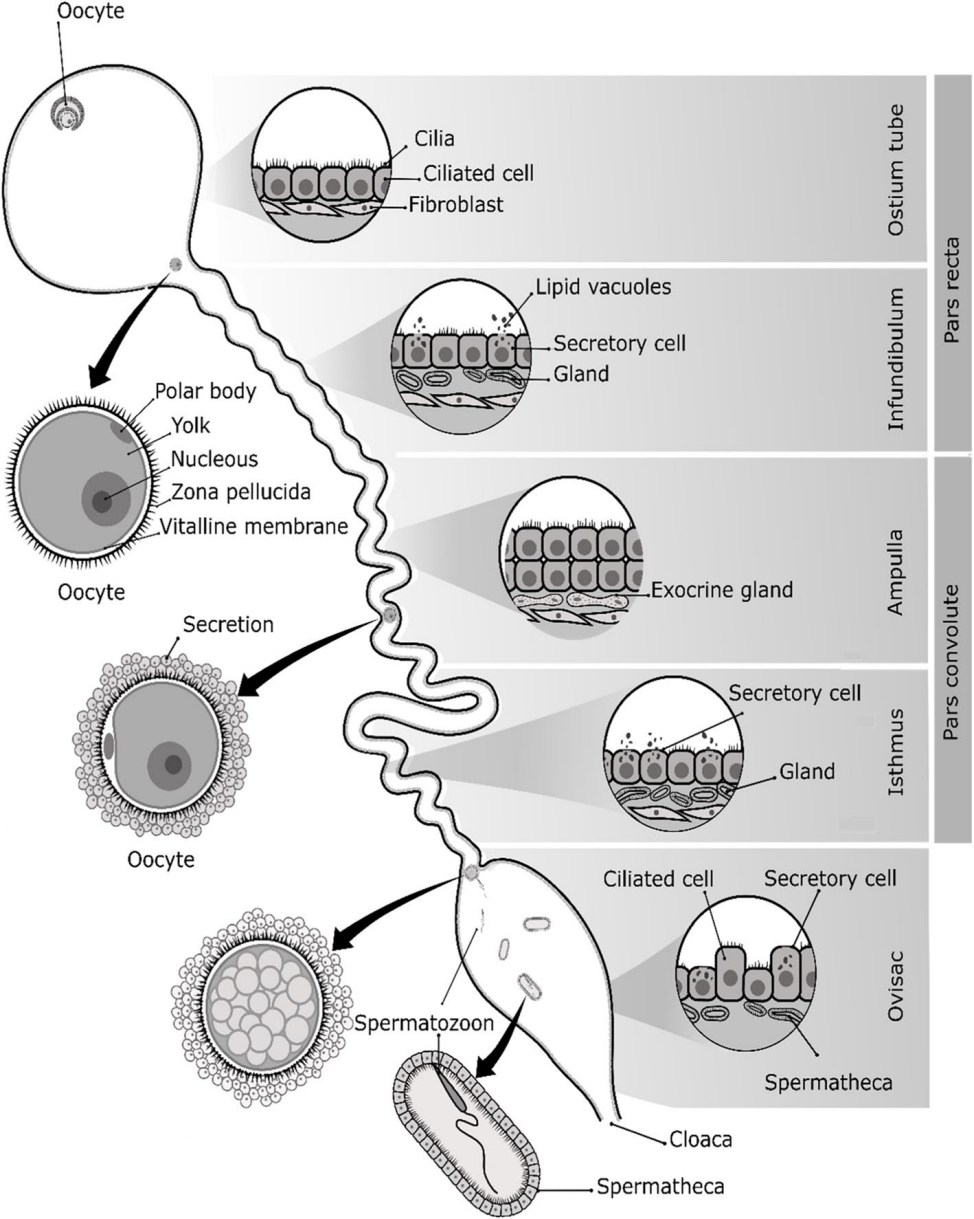
Oviductal regions of frogs. The oviduct consists of three regions: the infundibulum, the ampulla, and the isthmus. These conform with the “pars convolute” and “pars recta”. The “pars recta” nurtures and transports the oocytes to the ovisac. The ostium tube is the transition from the “pars recta” into the infundibulum. Generally, the ostium tube is composed of numerous cilia and is aglandular. The infundibulum is formed of ciliated cells, glands, and secretory cells with lipid-secreting granules. The ampulla is a highly secretory portion of the “pars convoluta”. The exocrine glands and epithelium secrete a jelly-like substance to surround and nurture the oocyte. The isthmus has mucosal folds, ciliated cells with short cilia, and ceramide-secreting cells. Finally, the ovisac stores the oocytes, and the spermatheca store sperm for fertilization

Ovulation is a different reproductive process in frogs: no follicular fluid is present, and the process of follicular rupture in the anurans is hardly comparable with that of mammals because there is no phase similar to the menstrual cycle [2]. The differentiation of the oogonia occurs in the cortex; these germinal cells form follicles surrounded by somatic cells and are located in the medulla [10]. A follicle is the morphological unit of the ovary that produces oocytes surrounded by follicle cells (theca and granulosa) [58]. The gonadotropin hormones stimulate the secretion of progesterone and testosterone in the ovarian follicles [59]. Luteinizing hormone (LH) stimulates the release of steroids in the granulosa cells that synthesize estradiol (E2) by the action of the enzyme aromatase. The different steroids which are synthesized mainly originate from follicular epithelium [55]. The female germ cells of frogs become oogonia, previtellogenic oocytes, vitellogenesis step oocytes, vitellogenic and postvitellogenic oocytes [60]. The oocytes are formed by a fine layer of follicular cells surrounding the theca cells, containing collagen, fibrocytes, yolk, and blood vessels [10]. In particular, the ovaries contain numerous previtellogenic (primary growth) and vitellogenic (secondary growth) oocytes [48, 56]. The oocyte development is divided into six stages according to size, yolk distribution, and pigment (Fig. 2) [61, 62]. During ovulation, these oocytes mature and differentiate in the ovaries to finally be released to the oviducts (Fig. 3) [46].

#### Stage I

In the premeiotic stage, the “previtellogenic oocytes” are transparent cells with a granular cytoplasm and a nucleus that takes up most of the cell center. In *Xenopus laevis*, the previtellogenic oocytes range from about 50 to 300 μm diameter [10, 61]. Oocytes of 250 μm diameter have been detected in *Xenopus laevis, Flectonotus pygnzaens, Gastrotheca ovifera*, and *Gastrotheca sp* [63]. During the vitellogenic phase, *Rana lessonae* and *Rana ridibunda* have oocytes of 50 to 300 μm diameter [64]. In *Rana tigrina* the size of previtellogenic oocytes is 107 to 430 μm and predominates until stage III [65]. The oocytes have Balbiani bodies, squamous follicle cells, and blood vessels. Particularly, the Balbiani bodies are only present during the early stages of oogenesis [10]. Balbiani bodies are a temporary site of an organelle composed of the endoplasmic reticulum, germplasm, messenger RNA (mRNA), mitochondria, Golgi apparatus, proteins, germinal vesicles, and vitelline bodies (Fig. 2, I) [56]. During this stage, the oocytes are located in somatic cell nests connected by intercellular bridges [66]. After the mitotic phase, the primary oogonia become the precursor cells of secondary oogonia [10]. In contrast, the primary oogonia are individual cells smaller (15 μm in diameter) than primordial germ cells and are surrounded by somatic pre-follicular cells [64]. In this stage, the oocyte is surrounded by a single layer of somatic cells that become follicle cells [61, 64]. It has been determined that primordial germ cells are large and filled with yolk platelets. Subsequently, primordial germ cells start mitotic divisions and the vitellolysis. Some species, such as *Xenopus laevis*, have colorless oocytes with transparent cytoplasm [10]. In others, such as *Rana lessonae*, the vesicle is filled with black pigment in diplotene oocytes [64].

#### Stage II

In this stage, the “early vitellogenic oocytes” measure approximately 300 to 450 μm in diameter in *Xenopus laevis* [10]. In *Flectonotus pygnzaens*, *Gastrotheca ovifera*, and *Gastrotheca* sp., oocytes can measure 250 to 500 μm [63], while *Rana lessonae* and *Rana ridibunda* have oocytes measuring 300 to 400 μm in diameter during vitellogenesis. Early oocytes at the leptotene, zygotene, and pachytene stages are formed during the first meiotic prophase. During the mitosis phase, the precursor cells remain connected by cytoplasmic bridges. At the end of pachytene, the cytoplasmic bridges disappear and each individual germ cell becomes enveloped by pre-follicular cells [64]. These cells form a group of somatic follicular cells and then become oocytes. Oocytes have an acellular nucleus around the yolk envelope and are previtellogenic [67]. During meiosis, the oocytes grow and accumulate yolk, turning opaque white in color [10]. The precursor molecule of the yolk is vitellogenin in oocytes. The yolk is composed of yolk platelets and fatty yolk; approximately 15% lipid content is found in yolk platelets in amphibians [10, 62]. This stage is characterized by the granular content of mucopolysaccharides and lipids in the oocyte cytoplasm [10]. Different enzymes fragment the yolk, which later provides nutrition to the embryos [58]. The peripheral cells comprise a monolayer of follicle cells that encases the oocyte. Subsequently, the oocytes develop the acellular vitelline envelope and take on an opaque white appearance (Fig. 2, II). The visible pigmentation of the oocyte cannot be seen at this stage due to low melanin synthesis. Generally, the vitellogenic oocytes comprise 45% of the population in stage II to VI [10]. For example, *Pelophylax ridibundus* has a large number of vitellogenic oocytes. It is common in the ovary of amphibians for digestion of the oocyte by its atrophied follicular cells that engulf and invade the follicle, to later degenerate and accumulate pigment [68].

#### Stage III

During stage III, the “midvitellogenic oocyte” reaches a diameter of 400 to 600 μm in *Xenopus laevis* [10]. In *Flectonotus pygnzaens*, *Gastrotheca ovifera*, and *Gastrotheca* sp., oocytes can reach 700 to 800 μm diameter [63]. *Rana lessonae* and *Rana ridibunda* have oocytes of 400 to 550 μm diameter [64]. Two important events occur in this stage: vitellogenesis and oocyte pigmentation [10]. Vitellogenesis refers to the phase during which the oocyte develops and produces yolk. The vitellogenin synthesis occurs in the endocytic vesicles of the oocytes. Therefore, oocytes accumulate large quantities of yolk protein in this stage. The yolk content increases by approximately 73% during oocyte development in *Xenopus laevis* [69]. On the other hand, *Flectonotus pygmaeus* produces few mature oocytes per breeding period; the oocytes accumulate a store of yolk and reach their largest size [63]. Oocytes are enveloped by two cellular layers and connective tissue [10]. Generally, melanosomes located in the cortical layer of the oocytes give them a dark brown or black color. The height of cubic follicle cells increases, and numerous blood vessels are developed on the oocyte surface. Finally, the follicles are composed of a diplotene oocyte surrounded by the granulosa cells and fine collagen fibers (Fig. 2, III) [64, 68].

#### Stage IV

Stage IV “midvitellogenic oocytes” have a size of 600 to 1000 μm diameter in *Xenopus laevis* [10]. In *Flectonotus pygnzaens*, *Gastrotheca ovifera*, and *Gastrotheca* sp., these oocytes measure 800 to 900 μm [63], and *Rana lessonae* and *Rana ridibunda* have oocytes of 550 to 700 μm diameter [10, 69]. The oocytes at this stage are full of yolk and pigment granules are observed [64]. The previtellogenic and early vitellogenic oocytes (431–646 μm diameter) have light but visible pigmentation in *Rana tigrina* [65]. The oocytes increase in number and size, and they protrude into the ovarian cavity [70]. In addition, the follicle attaches to the ovarian wall, and the oogonium begins to move from the nucleus to the centrosome of the germ cells [71]. Follicles constitute theca cells, granulosa cells, connective tissue, fibroblasts, and collagen fibers. The theca cells form part of the extracellular matrix that surrounds blood vessels, and fibroblasts [45]. The number of blood vessels increases in the theca cells surrounding the oocyte [10]. The theca cells are thick, separated, elongated, and have spindle-shaped nuclei. Theca cells synthesize and secrete estradiol, progesterone, and 17 hydroxyprogesterone (Fig. 2, IV) [72].

#### Stage V

The size of the “vitellogenic oocyte” ranges from 1000 to 1200 μm in *Xenopus laevis* [10]. *Flectonotus pygnzaens*, *Gastrotheca ovifera* and *Gastrotheca* sp. have oocytes measuring approximately 1000 μm in diameter [63]. *Rana lessonae* and *Rana ridibunda* have oocytes measuring 700 to 1000 μm in diameter [64]. Generally, the oocytes are medium-sized and uniformly pigmented. In the growth phase, the oocyte lacks distinction between animal and vegetal poles in *Rana tigrina* [65]. In addition, the follicular cubical cells are thicker and form different layers. The zona pellucida and microvilli are on the surfaces of the oocyte. The microvilli are extended to oocytes, melanin deposition increases, and the theca cells are maintained similarly. Finally, the preovulatory oocyte is surrounded by the cellular and acellular layer. Numerous blood vessels are located on the oocyte surface (Fig. 2, V) [58]. *Xenopus laevis* have nests that contain approximately 16 oocytes [73]. Another study found that this species forms a bag with about 20 lobe-containing oocytes at all stages of growth in frogs [74]. However, late pachytene oocytes are not found in cell nests and are principally surrounded by follicle cells. On the other hand, oogonia are characterized by an irregularly shaped nucleus. The oogonia are distributed in the germinal epithelium and form groups of cell nests of late oocytes [73]. Secondary oogonia are a source of a renewing stem germ cell population in the ovary. These oogonia form nests of leptotene-pachytene oocytes, and they are pigmented from dark brown to beige [75]. For example, in *Bokermannohyla ibitiguara* oocytes are black at the animal pole, and beige at the vegetal pole [76].

#### Stage VI

During stage VI, vitellogenesis has ended, and the growing oocytes have completed their development. The “postvitellogenic oocytes” finish their development, reaching 1200 to 1300 μm in *Xenopus laevis*, and they are pigmented brown to pale yellow [10]. In *Flectonotus pygnzaens, Gastrotheca ovifera* and *Gastrotheca* sp. mature ovaries have large oocytes measuring 1000 to 3000 μm in diameter, and the oocytes are yellowish-white [63]. In *Rana temporaria, Rana lessonae*, and *Rana ridibunda*, the oocytes measure 1000 to 2000 μm in diameter, and they achieve meiotic maturation before ovulation. *Rana tigrina* forms large yolky oocytes (1078 μm in diameter) with complete distinction between the animal and vegetal poles [65]. During oocyte maturation, the communication between granulosa cells and oocytes is carried out by the microvilli and follicle cells. The microvilli of the oocytes extend onto the surface of follicle cells, and the zona pellucida or vitelline envelope is formed around the oocyte. The vitelline envelope develops two different layers composed of glycoproteins in the oocyte [73]. The microvilli are located in compartments filled with the substance vitelline (Fig. 2, VI). During oocyte release, maturation-promoting factor (MPF) contributes to nuclear membrane breakdown [77]. LH and MPF are essential to the division of the primary oocyte, forming a secondary oocyte and the first polar body until fertilization. LH stimulates the follicle cells to synthesize and secrete progesterone, and progesterone activates MPF to mature the oocyte during ovulation [78]. Finally, the oocyte degeneration process is divided into four stages in *Rana temporaria, Rana lessonae,* and *Rana ridibunda*.

#### Oviduct

Ovoviviparity is a reproductive mechanism where eggs are retained inside the female. Then, eggs hatch into adult frogs after an incubation period in structures that are usually not involved in reproduction, such as the vocal sac [18], a dorsal sac [14], or the stomach [17]. Caecilians and salamanders have maternal nutrition through the proliferation of oviductal mucus cells. However, no amphibian species has developed a placenta or pseudoplacenta [79]. It has been shown that *Adelophryne maranguapensis* has an oviduct formed by a ciliated and glandular luminal epithelium [80]. *Lithobates catesbeianus* has a folded epithelium, numerous glands, and vacuoles that secrete glycoproteins and lipoproteins [11]. *Xenopus laevis* has oviducts 3 cm long that are divided into different segments based on morphology [81]. *Pelophylax ridibundus* secretes and releases a jelly-like substance from the oviductal glands by the action of the progesterone hormone [82], while androgens control the growth of the oviduct in *Euphlyctis cyanophlyctis* [83].

Anatomically, the oviducts are two wide, tangled tubes originating at the Müllerian duct. The oviducts extend from the heart to the cloaca to join the rectum and urinary conduit [13, 46]. These organs are suspended dorsally by the mesotubaria tube. In general, the oviduct is divided into three main sections: the pars recta (infundibulum), pars convoluta (ampulla and isthmus), and the uterus or ovisac [58]. The “pars recta” has the function of collecting the oocytes from the coelomic cavity after ovulation. Subsequently, the “pars convoluta” nurtures and transports oocytes to the ovisac. Finally, in the ovisac the oocytes are fertilized and develop during internal fertilization [84, 85] (Fig. 3). Histologically, the oviduct is formed by the mucosa (epithelium), serous tissue (connective tissue), and muscle tissue [11–13]. The mucosa contains ciliated cells, secretory cells, exocrine glands, and secretory granules. The submucosa is composed of connective tissue full of capillaries, and collagen fibers [13, 46]. Finally, the muscle layer is the thickest, formed by smooth and longitudinal muscle fibers between the connective tissue [12, 81].

The “pars recta” is a region of transition between the infundibulum (aglandular) and the “pars convoluta” (glandular). In this region, the epithelium is ciliated and cuboidal and contains few glandular cells. Secretory cells are more abundant than ciliated cells. The “pars convoluta” secretes a gelatinous layer that is important for egg transport. The secretions of the epithelium and glands are essential for spermatozoa penetration of oocytes [58]. The granules located in the epithelium contain vacuoles that secrete lipids, ceramides, and glycoproteins [11]. The infundibulum consists of a thin layer of folded and irregular mucosa. The essential function of the infundibulum is to receive the oocytes [85]. The epithelium is cuboidal with numerous ciliated cells. The submucosa layer is organized by connective tissue that contains blood vessels (Fig. 3). The myometrium or muscle layer of the oviduct is formed of circular smooth muscle fibers [86].

The “pars convoluta” is the largest region of the oviduct. The luminal epithelium is cuboidal and contains few ciliated cells. This region is highly secretory, and cilia integrate the mucosal layer to promote the transport of eggs through the oviduct. In particular, this region has tubular exocrine glands surrounded by thin layers of vascularized connective tissue. The jelly-like secretions increase during the passage of the oocytes into the oviduct [58]. Mucins or carbohydrates are involved in maintaining oocytes and eggs, as well as facilitating the recognition between oocytes and spermatozoa during fertilization [84]. In the ampulla, secretory cells and exocrine glands predominate, and secrete jelly-like layers onto the oocytes. These exocrine glands are positioned in the connective tissue. Generally, the exocrine glands are numerous and enlarged, which increases oviductal secretion [46], and the ciliated cells are distributed among the secretory cells. When mature oocytes emerge from the ovary, they are transported to the oviduct by ciliary movements [87]. The muscle layer of the ampulla is especially thin [12]. The isthmus is the last region of the oviduct, and it is formed by creases of mucosa with short cilia and ceramide granules, lipid vacuoles, and epithelial glands. Morphologically, the isthmus is composed of a folded epithelium with ciliated cells that are shorter than those found in the infundibulum. Here, the cilia cover the surface of the epithelial folds. The glands secrete lipoproteins and are located under the epithelium. These glands are filled with secretory vacuoles and numerous lipid granules. Similarly, the isthmus is coated by a serous membrane and a muscle layer. However, it is different from other regions because cilia entirely cover the folded mucous membrane on the epithelial surface (Fig. 3) [11].

#### Ovisac

The ovisac or uterus is located after the oviduct [88]. It connects with the cloaca and forms the oviductal sinus. The oocytes accumulate there for a short time [47, 89] to later be fertilized [12]. The ovisac is formed by a ciliated and non-ciliated folded epithelium, with exocrine glands full of secretory vacuoles that function as tubules for spermatozoa storage [81]. The oocytes are covered by jelly-like secretions during their passage through the ovisac [12], and even cloacal glands accumulate spermatophores [46]. A spermatophore is a capsule containing spermatozoa, especially in amphibians. The spermatozoa are stored in the oviductal glands, sperm storage tubules, simple tubular glands, or spermatheca located in the oviduct. In some species, the oviduct of frogs is formed by numerous spermathecae (Fig. 3). Histologically, spermathecae are formed by connective tissue, a circular layer of smooth muscle (myoepithelial cells), and some epithelial cells that never possess cilia [12]. Furthermore, the ovisac is different from the oviduct because it contains thick layers of connective and muscle tissue. That morphology is essential to support the intense contractions during oviposition [58].

#### Incubation of eggs in typically non-reproductive structures

**Fig. 4:**
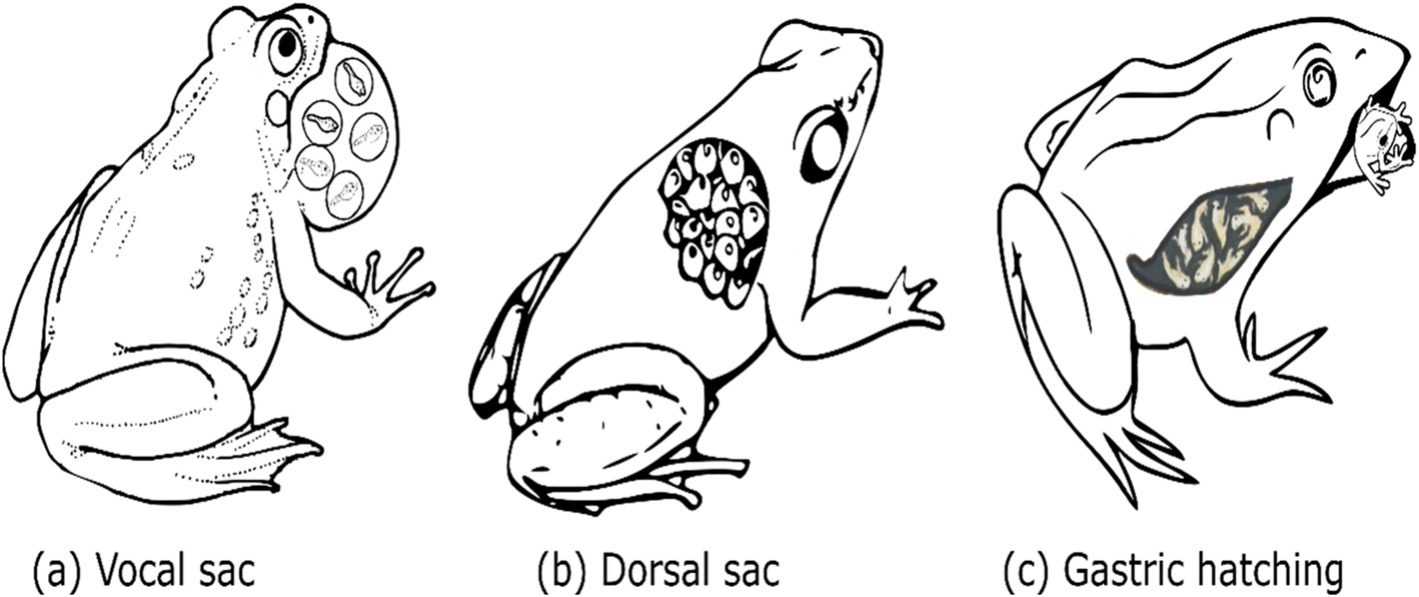
Typically, non-reproductive structures are used as oocyte incubators in some frogs. Larval development and metamorphosis can occur within the vocal sac, where frogs incubate the eggs and secrete nutrients for embryo development (**a**). The females carry the eggs on their back and provide maternally derived nutrients via yolk (**b**). Gastric hatching frogs ingest fertilized eggs, after which they develop in the stomach and emerge as juvenile frogs (**c**)

Some frogs house embryos in structures that are typically not involved in reproduction, such as the vocal sac [16], stomach [17], or morphologically adapted dorsal sac [14] (Fig. 4a). Larval development and metamorphosis can also occur within the vocal sac of males and can take approximately 8 weeks. The incorporation of eggs into the vocal sac permits the supply of food and oxygen to larvae through the epithelium. After fertilization, the frogs that incubate eggs in the vocal sac secrete nutrients to maintain embryo development. In addition, the epithelial cells of the vocal sac secrete a viscous fluid that is transported paracellularly between parent and larvae [18]. The juvenile individuals emerge from the vocal sac to the mouth through two slits located in the lower part of the mouth that connect the two cavities. *Rhinoderma darwinii* is a species in which tadpoles undergo complete metamorphosis inside the male vocal sac; development takes 52 days. The males pick up the eggs one by one with the mouth and introduce them into the vocal sac [16, 18].

Some species have a special sac for hatchling development. For example, *Cryptobatrachus remotus, Flectonotus pygmaeus*, and *Stefania ginesi* hold eggs in a sac formed by two flaps of vascularized and adipose skin tissue located on their dorsum [15]. The females carry the eggs on their back and provide maternally derived nutrients via yolk. For example, females of the genera *Flectonotus* and *Gastrotheca* keep the eggs in closed sacs that extend from the dorsum to the frog’s neck (Fig. 4b) [18]. In *Gastrotheca excubitor*, the embryos are surrounded by a highly vascularized membrane within bags, and the gills of hatchlings undergo gas exchange and nutrient transference [14]. In *Flectonotus pygmaeus*, the eggs are fertilized externally and immediately moved into the pouch to incubate [63]. Meanwhile, *G. riobambae* develops eggs in the bag for nearly 4 months [90]. In gastric-hatching frogs, the stomach is used as an incubation organ. The stomach expands, the epithelial membrane becomes thinner, and there is an increase in the apical microvilli. The columnar mucus-secreting cells are short, there are few mucus droplets, and oxyntic cells increase. The smooth muscle cells cluster circularly and associate with connective tissue [17]. Eight weeks after ingesting the eggs to incubate them in the stomach, the juvenile frogs emerge. This is an important example of how the stomach can be transformed from a digestive organ into a protective gestational sac [18] (Fig. 4c). For example, *Rheobatrachus silus* tadpoles and juvenile frogs inhibit the secretion of stomach acid while being incubated via the action of prostaglandin [17]. The stomach secretes drops of mucus and enzymes (pepsinogen granules) [91].

### Male reproductive system in frogs

#### Fat bodies and testicles

**Fig. 5:**
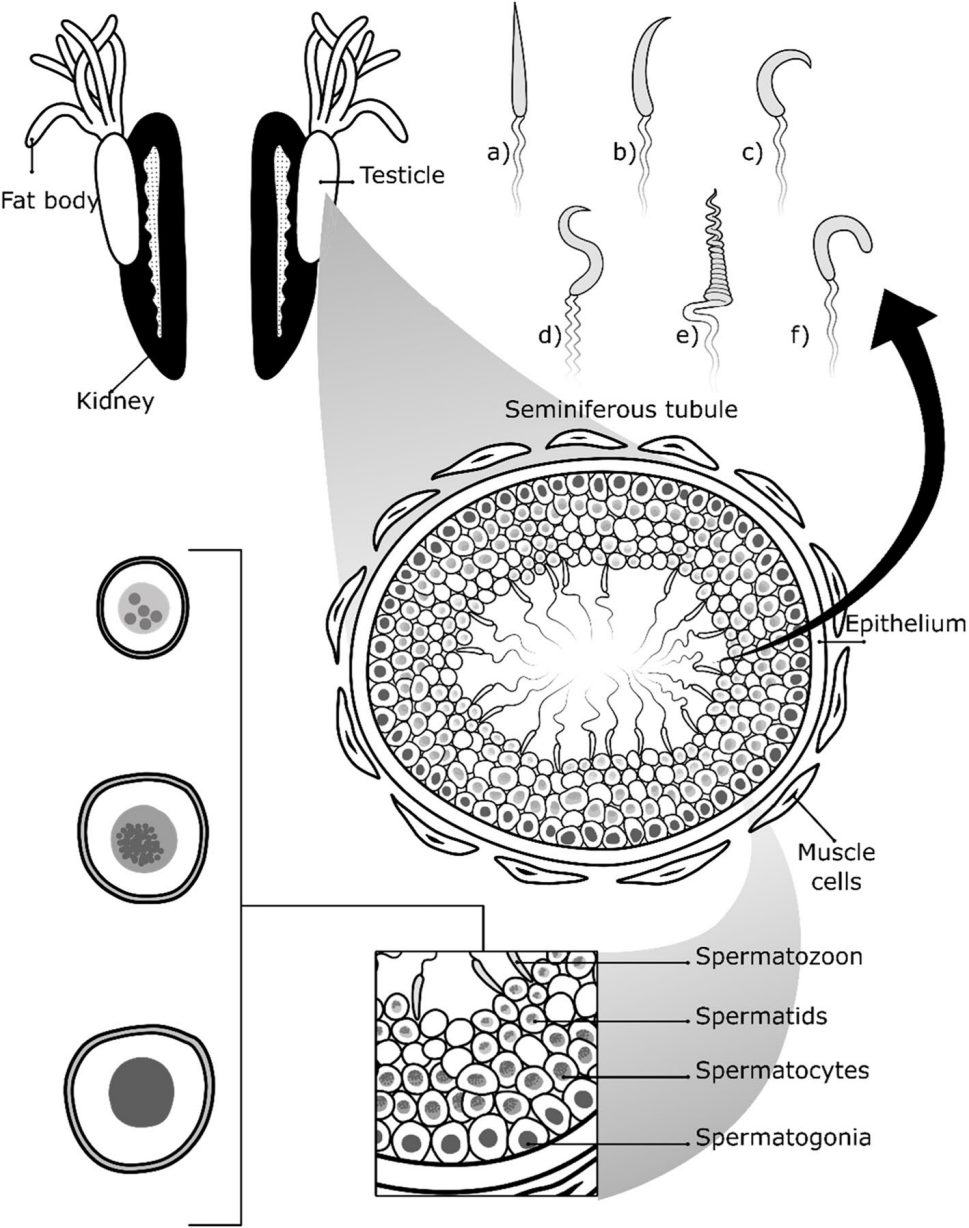
Male frog reproductive system. The fat bodies are above the testicles, both of which are located in the ventral region adjacent to the kidneys. The testicles consist of seminiferous tubules where spermatogenesis occurs, and interstitial space between the adjacent tubules. The seminiferous tubules are conformed by a seminiferous epithelium, connective tissue, and muscle cells. The seminiferous tubule houses the spermatogonia, spermatocytes, spermatids, and spermatozoa. Spermatogonia are usually large, voluminous, spherical cells. The spermatocytes have a smaller nucleus and differentiate into spermatids (long or round cells). Spermatids are located in the seminiferous tubule lumen and differentiate into spermatozoa. Spermatozoa morphology differs among frog species; (a) pointed head (*Odontophrynus cultripes*), (b) crescent-shaped (*Afrixalus frog*), (c) sickle-shaped head (*Rhacophoridae*), (d) S-shaped head (*Nyctibatrachus major*), (e) corkscrew-shaped head (*Xenopus laevis*), and (f) cane-shaped head (*Polypedates maculatus*)

In amphibians, the urogenital system consists of the testicles attached to fat bodies and accessory ducts [85]. They are located above the testicles and are whitish, yellowish, or orange in color. The testicles are located in the ventral region of the kidney in the abdominal cavity. The kidneys are closely bound to the testicles by the mesentery [92] (Fig. 5). These gonads are divided into a germinal compartment and an interstitial compartment. The germinal compartment comprises the seminiferous tubules, spermatogonia, spermatocytes, spermatids, spermatozoa, and Sertoli cells, while the interstitial compartment is composed of collagen fibers, blood vessels, Leydig cells, and connective tissue [18, 93]. Histologically, the testicles are composed of a network of seminiferous tubules, connective tissue, and the tunica albuginea (fine collagen fibers). The contractile cells in the tunica albuginea are peritubular myoid cells that form a single layer around the seminiferous tubules [94]. The albuginea tunica is a thin layer and has no pigment cells [93]. However, in some species, melanocytes are distributed in the albuginea tunica and the interstitium. Little is known about the function of pigmentation in frog testicles [2, 93]. Pigmentation of the testicles rarely has been observed due to the presence of melanocytes in the interstitium as described in *Physalaemus cuvieri* and *Physalaemus fuscomaculatus* [95]. The testicles of amphibians are usually lobular in shape. However, the shape of the testicles varies among species of frogs. For example, the Mexican leaf frog’s testicles are elongated and translucent during most of the year, but they become thicker and milky yellow during summer [92]. Fat body weight, plasma testosterone, and spermatogenesis vary with seasonality in *Rana perezi* [96]. It has been determined that the fatty bodies represent an anatomical structure necessary for transporting hepatic lipids to the testicle, with blood being the main channel between the liver and the fatty body. Acetyl-CoA, a lipid precursor in the fat body, varies during the reproductive cycle in *Rana esculenta* [97].

#### Spermatogenesis

Currently, there is limited information on the process of spermatogenesis in amphibians [4]. An essential characteristic of amniotes is that spermatogenesis takes place in cysts [98]. The cysts open to release spermatozoa during spermiation [99]. Regarding spermatogenesis: 1) spermatogonia are found in cysts, 2) the cysts are formed by germ cells and Sertoli cells [98], 3) spermiation does not occur in the periphery of the testicles, and 4) spermatogenesis in amphibians has generally been described as similar to the process in humans [100]. In amphibians, both prespermatogenesis and spermatogenesis occur. In prespermatogenesis, the gonocytes proliferate during the development of the testicles in tadpoles. During active spermatogenesis, spermatogonial stem cells inside the cyst proliferate and then subsequently differentiate into spermatozoa in adult frogs [92, 101]. Zirkin described nuclear morphology in spermatids and spermatozoa of *Rana pipiens*. In stage 1, the early spermatids have round nuclei, but during stage 2, there is a reorganization of the nuclear material of the spermatids, and the nuclei are of various sizes. In stage 3, mature spermatozoa contain elongated and cigar-shaped nuclei. Finally, in stage 4, mature spermatozoa have elongated nuclei and are round [101]. Sun classified spermatogenic stages in Korean frogs (*Rana dybowskii, R. nigromaculata,* and *R. rugosa*). First, in the immature stage (stage I), the cysts fill with spermatogonia and increase in number. In this stage, there are no spermatozoa in the seminiferous tubules. In early spermatogenesis (stage II), the spermatogenic cells appear in all development phases, including some spermatozoa. However, the seminiferous lumen is not formed yet. In addition, the spermatocytes and spermatids increase, while spermatogonia decrease. In late spermatogenesis (stage III) primary and secondary cells are present: primary and secondary spermatogonia, primary and secondary spermatocytes, spermatids and spermatozoa. The lumen of the seminiferous tubule has formed, but spermatozoa are not found in the lumen of the tubule. In spermiation (stage IV), the seminiferous tubule lumen is filled with spermatozoa, and only some gonadal cysts remain [102]. Pudney similarly classified spermatogenesis in nonmammalian vertebrates: primary spermatogonia, secondary spermatogonia, primary spermatocyte, secondary spermatocyte, primary spermatid, secondary spermatid, tertiary spermatid, and spermatozoon [103].

Chavadej classified spermatogenic cells into 12 stages based on nuclear characteristics and sizes in *R. catesbeiana*. The primary spermatogonia (stage I) constitutes a high proportion of the cell population in seminiferous tubules. The cells are large with a round or oval nucleus (10–13 μm) located in the basement membrane. Secondary spermatogonia (stage II) are round and small, with a nuclear diameter of 9–12 μm. The group of secondary spermatogonia is formed by two or four cells located in the basement membrane. Leptotene spermatocytes (stage III) are organized into groups of four cells with large (11–13 μm) round nuclei. In zygotene spermatocytes (stage IV), the nuclei become smaller than leptotene spermatocytes. Pachytene spermatocytes (stage V) have nuclei that are round and 10–12 μm diameter. In diplotene spermatocytes (stage VI), the nuclear diameter is 10–11 μm, and these cells are more numerous than pachytene spermatocytes. In diakinetic and metaphase spermatocytes (stage VII), the nuclear boundaries disappear, and there are few spermatocytes within the seminiferous tubules. Secondary spermatocytes (stage VIII) arise after the first meiotic division, and nuclear diameter becomes smaller. The early spermatids (stage IX) decrease within the seminiferous tubules; the nuclei are still round and have a diameter of 8–9 μm. In middle or round spermatids (stage X), the oval nucleus is reduced in diameter to 6–7 μm. In late spermatids (stage XI), the nucleus is reduced in size and begins to elongate. Finally, the cells move close together and group to the lumen of seminiferous tubules. The mature spermatozoon (stage XII) has elongated head and flagellum. The head of spermatozoa is united in the apical cytoplasm of the Sertoli cell, while their flagellum points toward the seminiferous lumen [104].

In summary, frogs’ annual spermatogenic cycles depend on seasonal environmental changes [45]. The process of proliferation and differentiation of germ cells (spermatogonia, spermatocytes, spermatids, and spermatozoa) occurs in testicles [3, 104, 105]. This process is controlled by gonadotropin hormones, testosterone, and other androgens [106]. Spermatogonia divide and give rise to secondary spermatogonia; these cells form cysts and are smaller. Spermatogonia are oval-shaped cells that give rise to spermatocytes (haploid cells). Subsequently, spermatocytes (smaller cells than spermatogonia) differentiate into spermatids. Finally, the spermatids, which have a spherical or elongated shape, result from the second meiotic division and differentiate into spermatozoa (Fig. 5) [3]. Finally, spermatids elongate and transform into spermatozoa through spermiogenesis. This process involves the development of a flagellum from the basal body [4]. The head of the spermatozoan is in contact with basement membrane, but its flagellum is directed toward the center of the cyst [105].

#### Seminiferous tubules

The testicles are formed by the seminiferous tubules, where spermatogenesis occurs. The epithelium of the seminiferous tubules consists of Sertoli cells organized into groups that form spermatocytes, or spermatogenetic cysts [85, 92]. Additionally, seminiferous epithelium contains cells filled with glycogen [107]. Germ cells and Sertoli cells form a hematotesticular barrier in seminiferous tubules to generate a microenvironment that is appropriate for developing spermatozoa [108]. Different cell types (spermatogonia, spermatocytes, spermatids, spermatozoids) are located in the germ tissue in the seminiferous tubule lumen. The seminiferous tubules are formed of smooth muscle cells organized in a cell sheath without blood vessels (Fig. 5) [109]. In most species, spermatogenesis depends on the temperature and photoperiod. These two variables are critical external factors that regulate the reproductive cycle in amphibians [110, 111]. After reproduction, the density of spermatogonia, spermatocytes, and spermatids decrease in seminiferous tubules [112]. During winter, the seminiferous tubules contain mostly primary spermatogonia, as occurs in *Pachymedusa dacnicolor* where spermatogenesis is absent [92]. During the rearing period of the frogs, the seminiferous epithelium is thick. *Leptodactylus chaquensis* has a mass of convoluted seminiferous tubules among the interstitium. Additionally, this species has a duct between the seminiferous tubules and the ductus deferens for the release of spermatozoa [109].

#### Sertoli and Leydig cells

The agglomeration of germ and Sertoli cells form cysts filled with gonocytes [113]. Sertoli cells are follicular, supporting, and sustentacular cells in the testicles; i.e., they act as structural support for germ cells [92]. These cells are attached to the basement membrane in the seminiferous tubules [114]. The cell cytoplasm envelops the germ cells which are organized in cysts [50]. The cells remain in contact with the tubular basement membrane through cytoplasmic projections [109]. It has been determined that connexin is expressed mainly in Leydig and Sertoli cells. This protein is important for spermatogenesis and can be involved in synchronizing germ cell maturation inside the cysts [115]. In addition, Sertoli cells are associated with residual spermatozoa located between the interstitial and germinal compartments of the connective tissue [50]. The interstitial tissue is located in the seminiferous structures and is formed by collagen fibers, blood vessels, Leydig cells, and immune cells [5, 103]. The seminiferous tubules are surrounded mainly by interstitial tissue and Leydig cells. These interstitial cells are essential for spermatogenesis since they synthesize testosterone and other androgens [94, 107]. The Leydig cells occupy approximately one-quarter of the interstitial tissue [116]. They are either detached or in groups near the blood vessels [117]. Leydig cells are round, oval, or elongated, with thin granular nuclei found adjacent to the seminiferous tubules in the testicle [85, 92]. The cells are located eccentrically with one to three nucleoli [118]. For example, Leydig cells are stored in cysts in *Lithobates catesbeianus* and bullfrogs have cells with a higher proportion of nuclei than cytoplasm [116]. The Sertoli cells in *Physalaemus cuvieri* are voluminous, elongated, and ovoid [119].

#### Spermatogonia

The gonocytes are spherical or slightly elongated cells that differentiate into secondary spermatogonia for the following reproductive season. The gonocytes are surrounded by somatic cells (Sertoli cell precursors), and are located in the seminiferous tubule periphery [92, 120]. In the testicles, two types of spermatogonia have been identified: primary spermatogonia and secondary spermatogonia. Primary spermatogonia are located on the periphery of the seminiferous tubule [92, 109]. Primary spermatogonia are the largest cells; they are rounded and closer to the seminiferous tubules than secondary spermatogonia (Fig. 5) [92]. Furthermore, primary spermatogonia are bulky cells of the germinal lineage associated with Sertoli cells [120]. Secondary spermatogonia are smaller than primary spermatogonia and spermatocytes [11, 109]. Secondary spermatogonia and primary spermatocytes have a spherical nucleus [108, 120]. Generally, primary and secondary spermatogonia are estimated to form in 6 to 10 days [120]. In addition, secondary spermatogonia are mitotically divided and grouped into cysts (32–64 cells per cyst) [109]. The gonadotropin hormones and androgens regulate spermatogonial multiplication [106, 121]. For example, *Xenopus laevis* have smaller primary spermatogonia (8.5 to 12.5 μm) than primordial germ cells [4], and in *Pachymedusa dacnicolor* primary spermatogonia are wrapped by stem cells [92]. Meanwhile, *Lithobates catesbeiana* has more primary spermatogonia than other cells [117]. In *Pelophylax ridibundus* the gonocytes are larger than the spermatogonial stem cells [120]. In *R. lessonae, R. ridibunda* and *R. esculenta* the primary spermatogonia are pale and dark, respectively. Spermatogonia are characterized by a round shape and pale, round, or bean-shaped nuclei [105].

#### Spermatocytes

Spermatogenic cells are organized into spermatocytes, and each spermatocyte may contain cells at the same stage of the spermatogenic cycle. Generally, each spermatogenic cell contains germ tissue with many spermatocytes, which are groups of germ cells in the same cytodifferentiation stage. For example, in *P. bedriagae* the testicles consist of seminiferous tubules and include germinal tissue with spermatocytes and spermatogenic cells at the same cycle stage, although the morphology of the spermatids can vary in the species; for example, in this species the spermatids are elongated, spherical, or round with a unique ciliary structure [122]. During meiotic prophase, spermatocytes increase their size to 1.8 times more than that of secondary spermatogonia [92]. Spermatocytes undergo two nuclear cell divisions during meiotic maturation (leptotene, zygotene, pachytene, diplotene, and diakinesis) [104]. Then, the germ cells are differentiated into primary and secondary spermatocytes [108]. The spermatocytes in zygotene and leptotene are very similar structurally [92, 104]. The primary spermatocytes are formed in the first meiotic division and the secondary spermatocytes are formed after the second meiotic division, and these secondary spermatocytes ultimately give rise to spermatids [11, 108]. The secondary spermatocytes have small nuclei and form a cytoplasmic lobe when stored within the Sertoli cells. Subsequently, these secondary spermatocytes come into contact with the Sertoli cells [108, 117]. The spermatocytes become aligned along the cyst wall during spermiogenesis and each cyst contains approximately 200 cells [92]. The cysts are an arrangement of cells containing many secondary spermatocytes [108]. Finally, the secondary division of spermatocytes forms spermatids, which then become spermatozoa. In *Leptodactylus chaquensis* the spermatocytes are associated with intercellular bridges [109]. In *Platycephala* the spermatocytes are active during 4 months [123]. *Pelophylax lessonae* and *Pelophylax ridibundus* have from eight to 162 spermatocytes (pear-shaped cells) per cyst [120]. In *Scinax fuscovarius*, spermatogenesis occurs in seminiferous loculi, a structure that is formed by germ epithelium organized into spermatocytes [124].

#### Spermatids

The early round spermatids undergo spermiogenesis (to become mature spermatozoa). Spermatids are round cells associated with blood vessels in the seminiferous tubules [109]. The cells have elongated nuclei, and the chromatin is condensed. The young spermatids have larger cytoplasmic and nuclear volume than mature spermatozoa. The cytoplasm of the spermatids is lobular and extends from the head to the flagellum [117]. The cells are connected by cytoplasmic material and are formed of mitochondria, microtubules, accumulated glycogen, and lipid droplets [120]. The spermatids are released into the lumen of the seminiferous tubule after the rupture of the cysts [117]. The spermatid’s cytoplasmic lobes or residual bodies are degraded in the Wolffian ducts [92]. The Wolffian ducts are also storage sites for spermatozoa that are not yet capable of fertility [117]. In *Lithobates castebeiana*, spermatocytes are thicker and slightly more abundant in the seminiferous tubules, and some spermatids may even be present in the cloaca [109, 117]. Primary spermatogonial cysts are associated with Sertoli cells located close to the tubular lumen in *Leptodactylus chaquensis* [109]. *Lithobates castesbeiana* have seminiferous tubules with numerous spermatids in the tubular lumen. Spermatids are small, round cells near the cyst wall in *R. lessonae, R. ridibunda*, and *R. esculenta* [105]. In *Lithobates castebeiana*, the round spermatids are in contact with Sertoli cytoplasm, but this disappears later in development [117].

#### Spermatozoon

Amphibians can have a continuous or discontinuous reproductive cycle. In a continuous reproductive cycle, spermatozoa production is constant all year round until the cold season [45]. In the discontinuous cycle, the spermatozoa are present in a season delimited by the year [112]. Sperm production can be restricted to certain periods or seasons of the year [111]. *Rana temporaria* contains spermatozoa before winter [123] whereas in *Pelophylax kl. esculentus* there are degenerate spermatogonia in winter [106]. Frog spermatozoa differ among species in the shapes of head and flagellum [5, 6]. The head is formed by an acrosome and nucleus [120]. The spermatozoon enters first with its acrosomal tip to fertilize the oocyte. The flagellum has microtubules necessary for tension, mitochondria to give energy, and cilia for movement [125]. The movement of the flagellum and spinning of the head facilitate transport through a viscous environment by spinning up to three times per second [126]. The flagellum, the acrosome, and the midpiece of the spermatozoon contain glycogen, which is important for motility [127]. Spermatozoon head shapes include thick crescent shapes (e.g., in *Philautus* and *Afrixalus* frog) [6], corkscrew (*Xenopus laevis*) [5], pointed (*Odontophrynus cultripes*) [128], S-shaped (*Nyctibatrachus major*), sickle-shaped (*Rhacophoridae, Polypedates leucomystax*), rod-shaped (*Polypedates maculatus*) [6], straight (*Rana mutus*), and filiform (*R. megacephalus*) [5] (Fig. 5). *Polypedates leucomystax* [129] and the genus *Nyctibatrachus* have thick heads [6], and *Rhacophorus dugritei* have very thin heads [5]. *Rhacophorus malabaricus* does not have a coiled head [6].

Other species acquire their flagellum at the end of the spermatogenic process and have a thick or thin flagellum. *Xenopus laevis* first forms the flagellum and later the acrosome [4]. *Philautus and Afrixalus, Rana beddomei* [6], and *R. omeimontis* [5] have a thin flagellum (0.2 μm). However, the spermatozoon size does not seem to relate directly to the mode of reproduction. Green tree frogs, *Rhacophorus arboreus*, genus *Chiromantis*, and *Rhacophoridae, Polypedates leucomystax*, and *Rhacophorus schlegelii* have a thick flagellum that coils and uncoils to be able to fertilize by moving in a foam nest [5, 6, 125, 126]. *Pelophylax leucomystax* has a thick flagellum with two axonemes and many microtubules [129]. *Pachymedusa dacnicolor* has rigid fibers coiled into a spiral. In the proximal portion, it has mitochondria, and the flagellum is contractile [92].

## Conclusions

This review describes some basic but comprehensive schematics of the frog reproductive system. Due to the limited information on the reproductive system of species of the family Ranidae, this manuscript may contribute to a broader understanding of Rana’s reproductive biology. Furthermore, such information can be helpful for a morphological, histological, and physiological comparison of the reproductive system of frogs with other groups of the amphibian class since frogs are considered suitable biological models to study their reproductive biology and behavioral modes. Therefore, it is essential to know the functions of each reproductive organ in male and female frogs.

## Data Availability

Not applicable.

## Abbreviations

LH: Luteinizing hormone
E2: Estradiol
mRNA: Messenger RNA
MPF: Maturation-promoting factor
